# An RFID-Based Sensor for Masonry Crack Monitoring

**DOI:** 10.3390/s18124485

**Published:** 2018-12-18

**Authors:** Massimo Donelli

**Affiliations:** The Department of Information Engineering and Computer Science, University of Trento, 38100 Trento, Italy; massimo.donelli@unitn.it; Tel.: +39-0461-28-2063

**Keywords:** long range RFID, wireless sensor, modulated scattering technique (MST)

## Abstract

A radio frequency identifier (RFID) tag sensor for the real time monitoring of cracks in civil engineering building is presented in this work. The RFID tag is equipped with a piezoelectric sensor able to detect small movements of crack in order to prevent collapses of buildings or civil engineering structures. The information is delivered by using the modulated scattering technique (MST) which permits to obtain high operative ranges. The sensor is passive, the power supply is provided by means of a suitable rect-antenna and a Chockcroft-Walton multiplier circuit powered by means of the impinging interrogating electromagnetic wave. A system prototype, operating in the X band at 10 GHz, has been designed fabricated and experimentally assessed in a realistic scenario obtaining promising results.

## 1. Introduction

The structural integrity monitoring of civil engineering structures such as building, bridges, roads and dams is a topic of great interest [1,2,3,4], the recent collapse of Morandi’s bridge in Genoa, Italy it is an example of the need for efficient monitoring systems able to prevent sudden collapse. Moreover, after natural disasters such as earthquakes, floods, and landslips, the assessment of the structural integrity of civil engineering structures is of crucial importance to simplify the rescue operations, reduce the financial losses and save lives. Current monitoring methodologies are based on the visual inspection of the damaged structures or the analysis of samples obtained with coring. The visual inspections, is usually made by firefighters or expert structural engineers. Visual inspections are complex and dangerous especially after earthquakes, since the structures can suddenly collapse or they could be not easily reached due to rubble. The probing procedures based on coring could cause further damages or the structure collapse at worst. Another simple crack monitoring procedure widely used by construction companies and bricklayers is the use of glass crack width rulers. This method makes use of a small piece of glass fixed across a crack Small movements of the crack flaps break the glass and indicates a possible collapse risk. The glass crack width rules are widely used because it is cheap, effective and simple. The only disadvantage is that it requires a visual inspection to identify a break, and as stated above the visual inspection could be dangerous or impractical. Recently monitoring techniques based on microwaves [5,6,7,8,9,10,11], have generated a great interest in the field of structural integrity monitoring of materials and the detection of internal structure defects or anomalies without interfering with their integrity or functionality. Microwave techniques retrieve information concerning the material characteristics from the reflected electromagnetic waves by using suitable inverse scattering algorithms. These techniques despite their effectiveness are not suitable for real-time monitoring since they require too much high computational resources and time. Radio frequency identifier (RFID) is a well known technology which can be useful for monitoring applications. RFID systems have been initially developed for identification purposes and recently they have become an interesting alternative solution as sensing probe [12,13,14]. An RFID system is based on a tag which demodulates a low frequency interrogating signal provided by the reader. In particular an RFID tag transmits data to the reader introducing a modulation on the back scattered wave by means of a proper variation of the load impedance of the antenna tag [15,16,17,18,19,20,21,22,23]. The communication between tags and reader is done by means of inductive coupling with a limited operative range of a few centimeters. RFID tags are quite cheap because they are batteryless, they are activated by absorbing a portion of the electromagnetic wave power provided by the reader, long life and they do not require maintenance. Some commercial passive RFID tags are provided with external sensors and they are able to provide an operative range over 1 m (an example are the tags produced by the Farsens company, www.farsens.com). However despite the good performances of such tags, they are quite expensive and they cannot be further miniaturized since they operate at 900 MHz, a frequency band which requires antennas of big dimension with respect to microwave frequency bands, despite recent advances RFID systems are still not enough efficient for real time monitoring applications [16,17,18]). A valid alternative to RFID systems is the modulated scattering technique (MST) [24,25]. MST probes are flexible, low cost, and with high communication range since they can operate at microwave frequency bands. The pioneering work presented in [26,27] demonstrated the possibility of using a modulated scattering system to transmit data up to kilometers by using a limited amount of power, below one watt, at the reader. MST sensors operate with the same principles of RFID tags for this reason they can be easily integrated with limited hardware modifications in an existing RFID systems. Recently MST probes demonstrated their potentialities to monitoring the chemical properties of concrete and consequently the structural integrity of civil engineering structures [28], moreover they demonstrated their efficacy as real time continuous monitoring system for environmental parameters [29,30] and other interesting applications [31,32,33,34,35,36,37,38,39]. In this work a crack width ruler based on the MST technique, and able to operate at 10 GHz, is proposed and assessed in realistic scenarios. In particular, the proposed MST crack width ruler is able to monitoring the evolution of crack on in engineering structures enabling a safe remote inspection. The proposed MST crack detection sensor can be used to remotely inspect the structural integrity of buildings, bridges, roads by using a suitable reader placed far away from the dangerous area. The work is organized as follows. Section 2 reports a detailed description of a monitoring system and the guideline to maximize the performances of RFID/MST sensor tags. Section 3 reports on the preliminary experimental results obtained with a set of MST tags prototype that were assessed in a real scenario. Finally Section 4, provides conclusions and some points for future research.

## 2. System Description

**Figure 1:**
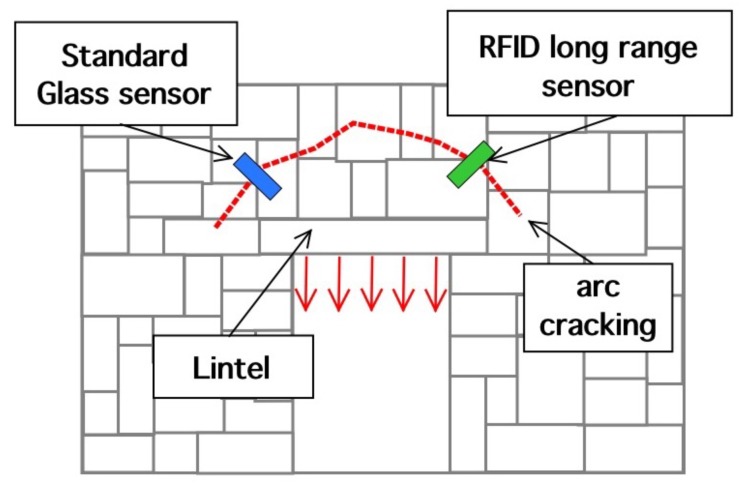
Problem geometry.

**Figure 2:**
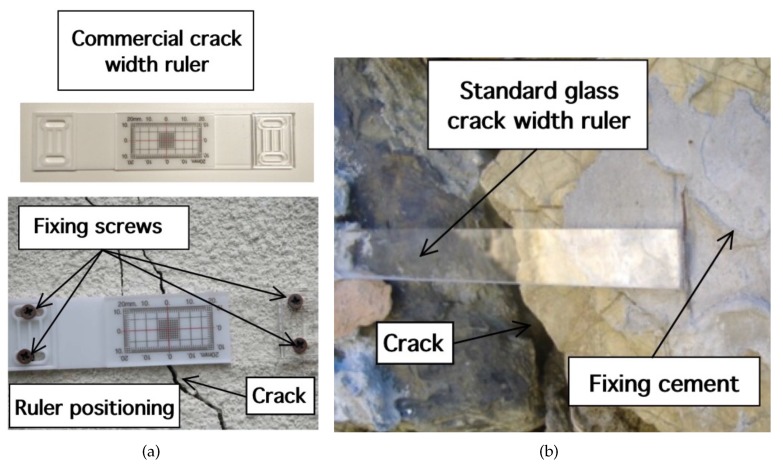
Crack monitoring rulers, (**a**) commercial and (**b**) standard glass ruler.

**Figure 3:**
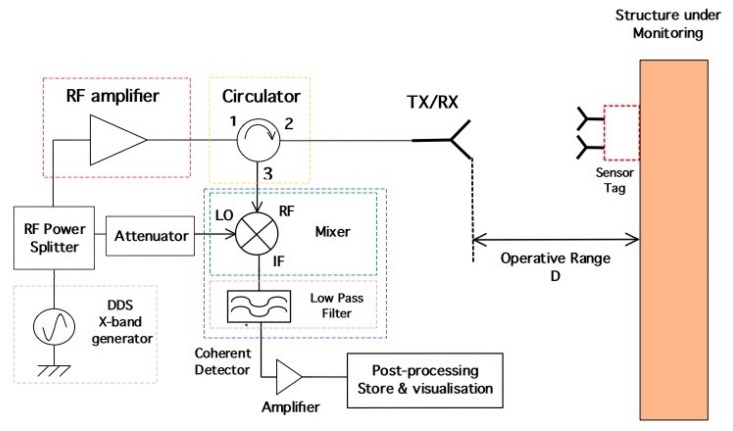
Reader schema.

This section is aimed at the description, design and optimization of the MST systems. The problem geometry is reported in Figure 1, where a damaged civil structure with a crack have to be monitored in order to prevent collapse. In particular the damaged reported in Figure 1, consists of an arc cracking placed just above the lintel, and it is particularly dangerous. To monitor such kinds of cracks, two devices are usually considered, the first one is a commercial crack ruler, shown in Figure 2a, it consists of two sections that can slide each other, in the middle there is a reference. When the crack flaps move, the displacement can be measured and stored. The second device, in Figure 2b, is a standard glass ruler. The proposed MST measurement system schema shown in Figure 3 consists of a reader aimed at communicating with a remote tag placed far away at a distance *D* called operative range. The tag is equipped with a piezoelectric sensor able to detect small movements of the cracks. The reader not only generates the electromagnetic wave, which impinges on the MST tag, but it also collects the back-scattered electromagnetic wave, reflected back by the probes, which carries the information. The MST tag is composed by two rectangular patch antennas, a power units consisting of a recantenna and a Cockcroft-Walton voltage multiplier, a variable controlled oscillator, an electronic switch, two resistive loads, a high sensitivity flexible piezo film probe and a charge amplifier. The impinging electromagnetic wave generated by the reader, provides the power to the charge amplifier, the VCO and the electronic switch. The switch connect the antenna tag toward two different loads. The variation of impedance produces a low-frequency modulation on the reflected electromagnetic wave. The low-frequency modulation carries the information, and it can be easily read at the reader output. A good MST system design permits maximizing the communication range *D* [25]. To maximize the operative range *D* the following radar equation, under the hypothesis of free space and far-field conditions, must be considered [40]:

(1) $$D=\frac{1}{2}{\left(\frac{P_{tx}\cdot G_{tx}\cdot \lambda^{2}\cdot A_{probe}\cdot G_{probe}\cdot ME\left(Z_{1},Z_{2}\right)}{{\left(4\pi \right)}^{2}\cdot P_{rx}}\right)}^{\frac{1}{4}}$$

where $A_{probe}$ and $G_{probe}$ are the antenna tag aperture cross-section and gain [41]. $P_{tx}$ and $G_{tx}$ are the power of the sinusoidal generator and the reader receiving antenna gain, respectively. $P_{rx}$ is the minimum detectable power, and it depends on the receiver sensibility, λ is the wavelength of the electromagnetic wave generated by the reader. The quantity $ME\left(Z_{1},Z_{2}\right)$ depends only by the two resistive loads and it is called modulation efficiency:

(2) $$ME\left(Z_{1},Z_{2}\right)=\frac{4Re\left\{Z_{probe}\right\}{\left|Z_{2}-Z_{1}\right|}^{2}}{{\left|Z_{probe}-Z_{1}\right|}^{2}\cdot {\left|Z_{probe}-Z_{2}\right|}^{2}}$$

where $Z_{1}$ and $Z_{2}$ are the two loads connected to the electronic switch and $Z_{probe}$ is the antenna tag impedance. The modulation efficiency ranges between 0≥ME and ME≤4. Considering Equation (1), it is quite evident that the only way to improve the communication range *D* is only to act on the values of the two loads $Z_{1}$ and $Z_{2}$ or to modify the antenna tag impedance $Z_{probe}$. The other parameters are fixed. To obtain the maximization of the $ME\left(Z_{1},Z_{2}\right)$, the values that maximize the modulation efficiency are $Z_{1}=0$ and $Z_{2}=Z_{probe}^{*}$.

### 2.1. Reader Description

The reader schema shown in Figure 3 is a monostatic radar configuration [40] consisting of a direct digital synthesis (DDS) X-band generator with an accuracy of about 0.1 Hz and a power of $P_{TX}=0$ dBm. The DDS generates an interrogating electromagnetic wave aimed at provide power to the MST tag and to carry the information reflected back to the reader by the MST tag. A microwave T-junction power splitter is aimed at provide the reference signal to a double balanced mixer (model PE86X10003 by Pasternack Enterprise, Irvine, CA, USA). The mixer and the reference signal represents the receiving section, which is a coherent homodyne detector [42,43]. The band base signal at the output port of the mixer is filtered by means of a low-pass stepped microstrip impedance filter, aimed at remove the high harmonic contributions then a suitable elaboration unit provides to post process and visualize the information provided by MST probes. The use of a homodyne detector is mandatory to use the measurement system in complex scenarios [25,29]. A circulator (model PE8403 by Pasternack Enterprise) permits the transmission and the reception of an electromagnetic wave and at the same time by using only one antenna, with a strong cost and dimension reduction of the reader structure. The transmitting/receiving antenna is a helical antenna which assure a good circular polarization. The considered helical antenna is the same reported in [28] and it was designed to produce a left hand circular polarization (LHCP). Circular polarization has been chosen in order to prevent possible misalignments between the reader and the MST tags. It has a gain of $G_{TX}=12.51$ dBi, a main beam aperture of $\Theta_{3dB}=20$ in the frequency range (9–11.5) GHz. A matching transformer with a ratio 1:4 has been considered to match the antenna impedance with the other commercial devices of the reader, characterized with a standard impedance of $Z_{0}=50$ Ω.

### 2.2. Tag Description

**Figure 4:**
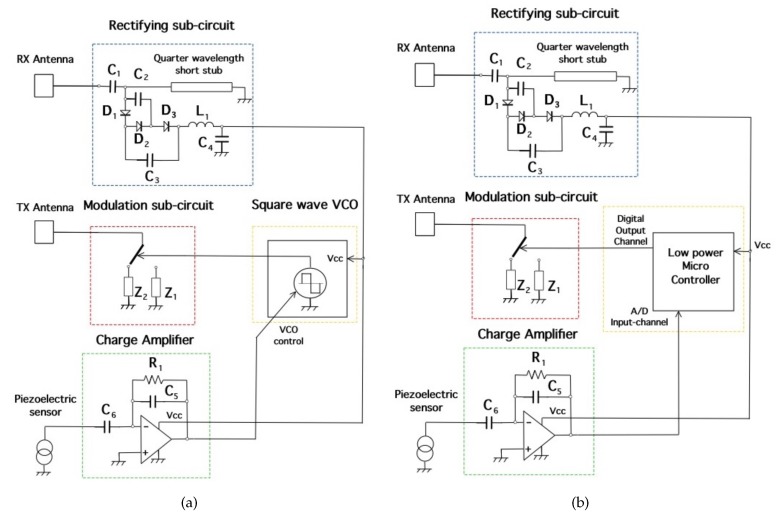
Tag schemas. Tag equipped with (**a**) VCO and (**b**) Microcontroller.

**Figure 5:**
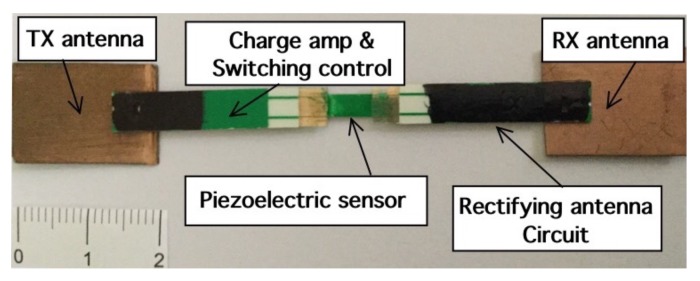
Tag photo.

**Figure 6:**
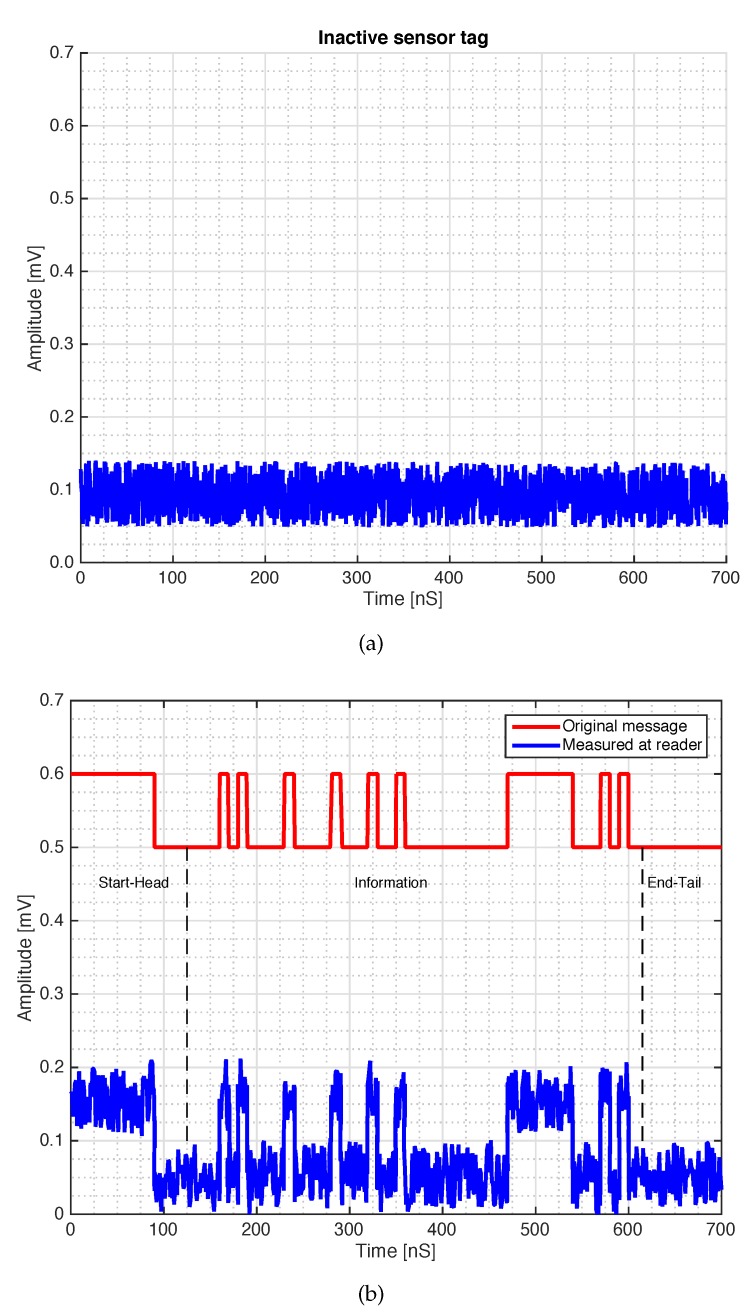
Signal detected at the reader output (**a**) inactive and (**b**) active sensor response. The signal has been generated with a MST tag equipped with a microprocessor to implement the EM4102 protocol.

This section describes the design of MST crack ruler sensor. It consists of two rectangular patch antennas printed on a dielectric substrate, namely Arlon 25N $\varepsilon_{r}=3.38$, tan(δ)=0.001, thickness t=0.8 mm. The transmitting/receiving patch antennas are centered af $f_{w}=10$ GHz, their dimensions are w=10 mm, h=8 mm and the feeding line is a microstrip of width $w_{0}=1.2$ mm. The receiving antenna is connected to a rectifying circuit consisting of a Cockcroft-Walton multiplier enhanced with a quarter wavelength short stub as in [20]. The efficiency of the rectifying subcircuit was between 55% and 60% . The rectifying circuit is aimed at supply the VCO, the MOSFET electronic switch, and the charge amplifier aimed at properly convert the signal provided by the piezoelectric probe into a measurable quantity. The rectifying subcircuit is equipped with a tank capacitor $C_{t}=47$ μF. The core of MST tag prototype is the piezoelectric transducer (LDT0-028K from Measurement Specialities company, Yumpu, Switzerland) a 28 μm thick piezoelectric polymer film, laminated to a 0.125 mm polyester substrate. The transducer is equipped with crimped silver contact. When the piezo film is displaced from the neutral position, bending creates high strain within the piezo-material and consequently high voltages are generates. The device data sheet shows that a displacement of only 2 mm produces a voltage of about 7 V. A charge amplifier [44] is mandatory to obtain open-circuit voltage sensitivity. The signal provided by the charge amplifier control the frequency of the VCO which can vary in a range from 1 KHz up to 4 KHz for a maximum displacement of 6 mm. Schema and photo of the MST tag prototype are reported in Figure 4 and Figure 5 respectively. The MST crack ruler operates as follows: the variable controlled oscillator or the microcontroller change their working frequency considering the data of piezoelectric flexible sensor aimed at detect the crack movements. The data are transmitted by changing the loads of the tag antenna using the electronic MOSFET based switch [29]. MOSFET-based switches offer the best performances [25], leading the ME nearly to the maximum theoretical upper. The transmitting antenna, loaded with different resistive loads, reflect back the impinging electromagnetic wave and add the information by means of the electronic switch [41]. The backscattered wave is received by the reader, the data are retrieved with the homodyne receiver and stored by mean of a suitable control unit. Another version of the MST tag is equipped with a low power microcontroller able to convert the analogic signal provided by the charge amplifier into digital data and send them to the switch by using a Manchester modulation and the same protocol adopted by a standard RFID system, the EM4102 protocol. The use of EM4102 protocol allows an immediate integration with commercial and RFID systems, databases and RFID resources. The protocol is organized as follows: the first ninebits at logical state one represents the head of the data string. Then, the head is followed by 10 groups of four data bits and one even parity bit. Finally, four bits of a column parity (even) and a stop bit (zero) are used as tail. Figure 6 reports an example of the data structure organized considering the EM4102 protocol and obtained with a MST tag equipped with a microcontroller. Concerning the power consumption, the tag equipped with the VCO requires a total power of about 30 μW, while the tag equipped with the microcontroller requires about 50 μW, a power comsumption similar to the device reported in [20], the microcontroller alone, requires 20 μW. In particular Figure 6a,b show the signal received at the reader for an inactive and active MST sensor response respectively. Since the considered tags have been designed to operate in indoor as well as in outdoor scenarios, they have been assessed at different temperatures. In particular a climatic chamber (ANGELATONI DY1200 with a temperature and humidity range of −40 °C < T < 180 °C and 10 % < H < 98 %) the climatic chamber has been kindly provided by the EMC company, Genoa, Italy. The results showed that the tag equipped with the microcontroller are quite insensitive to the temperature variations, while the tag equipped with the VCO is subjected to frequency shifts due to temperature variations. A calibration curve has been obtained for the tag equipped with the VCO to compensate for the errors.

## 3. Experimental Assessment

**Figure 7:**
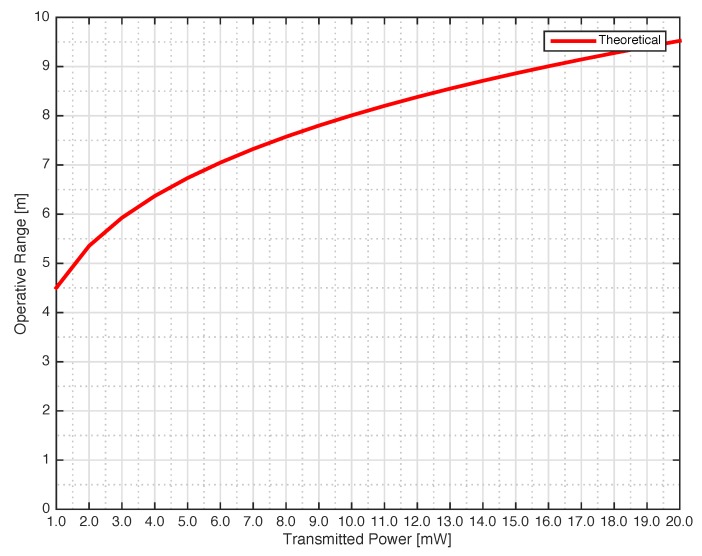
Assessment of the operative range vs. transmitted power in controlled enviroment.

Before starting the assessment of the MST system, the theory described in Section 2 has been assessed in a controlled environment. In particular, the operative range *D* versus the transmitted power was measured. The operative range was considered valid when the MST tag signal was correctly received a hundred times by the reader. Concerning the system parameter reported in relation (1), they are $G_{tx}=17$ dBi, $G_{probe}=6$ dBi are the reader and tag antenna gains respectively. The minimum detectable power at the receiver is $P_{rx}=-110$ dBm. The obtained modulation efficiency ME=3.3 was very close to the upper theoretical limit. The transmitter power $P_{tx}$ was changed in the range 1 mW $<P_{rx}<20$ mW with a step of 1 mW thanks to a programmable DDS generators able to change its output power. The capabilities of the MST system to correctly retrieve the signal have been assessed considering an MST tag placed in a line of sight (LOS) at different distances from the reader for different transmitting powers. The operative range *D* between the reader and the tag was accurately measured by using a commercial laser telemeter (Bosh PLR40C with an accuracy of ±2 mm). The obtained data are shown in Figure 7, a maximum operative range D=9 m has been obtained with a $P_{tx}=20$ mW, the obtained operative range is enough to provide a safe remote inspection in most real scenarios. As it can be noticed in Figure 4a,b the tag sub-circuits are directly connected with the rectifying circuit without a power supply stabilization circuit. The tag activation is dependant on the distance between reader and tag. The operative range can be then estimated by using the data reported in Figure 7. It is worth noticed that the operative range can be further improved by increasing the $P_{tx}$ of the generator.

**Figure 8:**
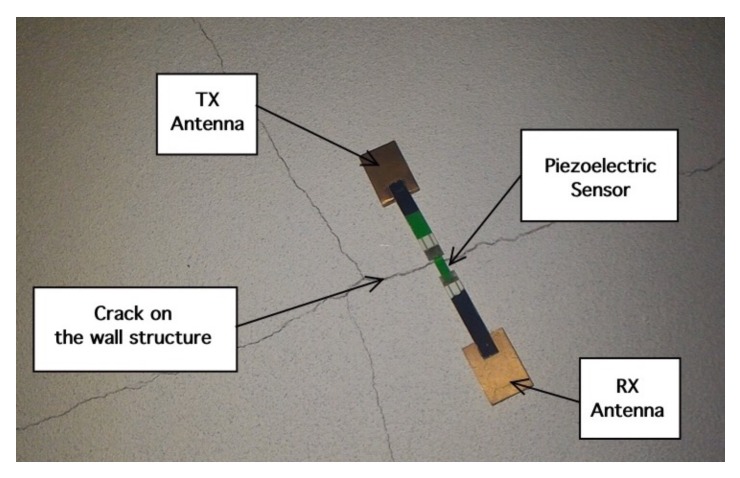
Photo of the crack and the tag placed on position B North side of the building.

**Figure 9:**
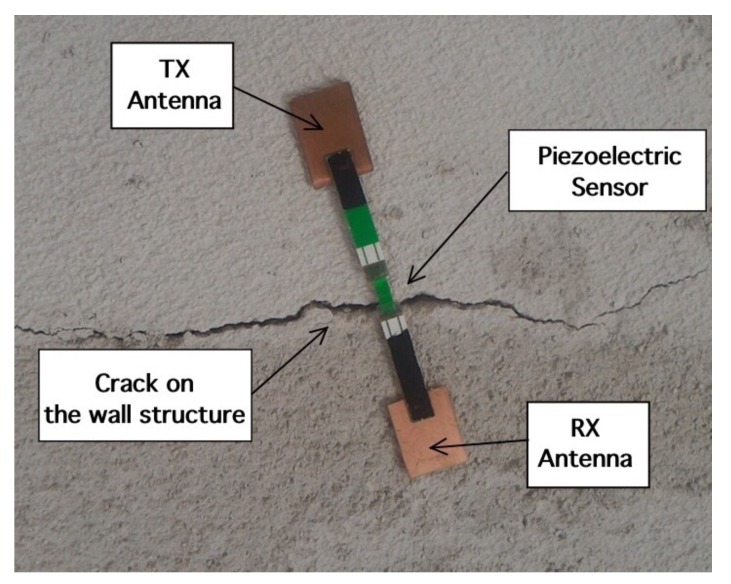
Photo of the crack and the tag placed on position A West side of the building.

**Figure 10:**
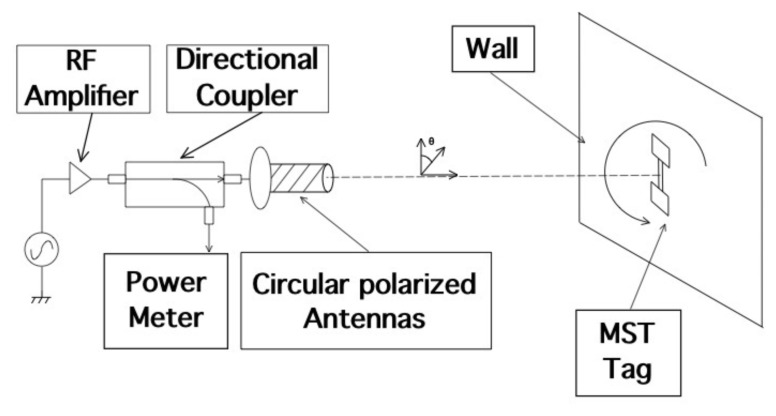
Assessment of the tag received signal vs. tag orientation. Experimental set-up.

**Figure 11:**
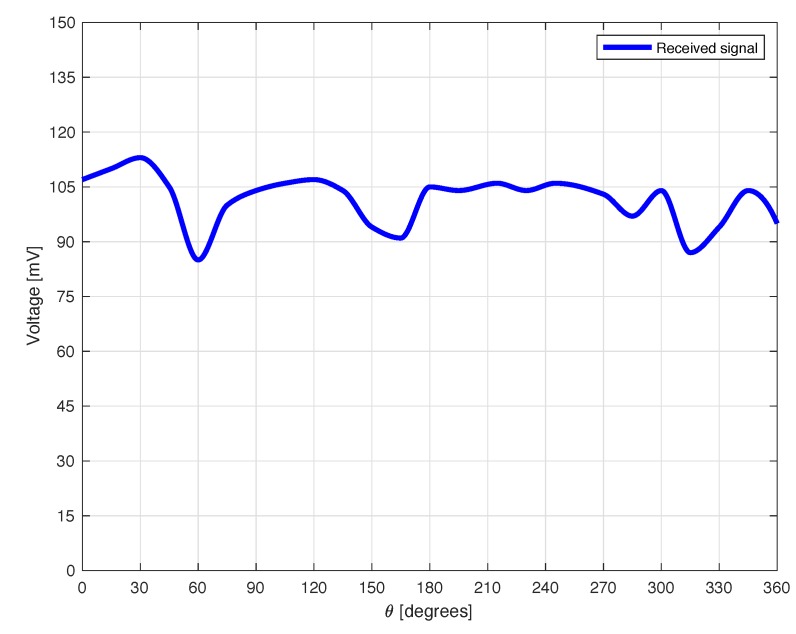
Assessment of the tag received signal vs. tag orientation. Measured signal vs. tag orientation.

**Figure 12:**
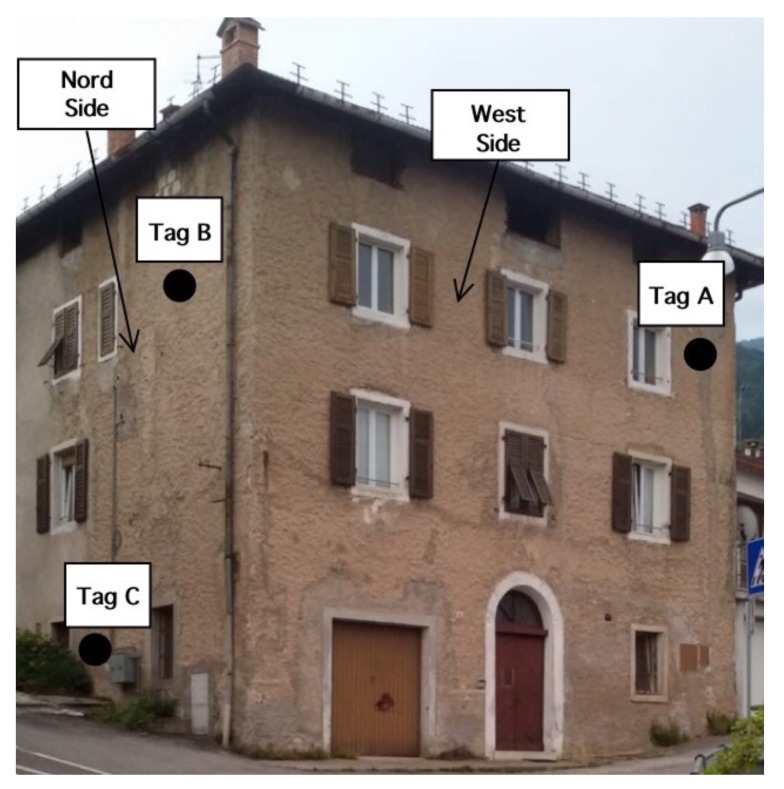
Photo of the considered experimental scenario, a 110 years old building under renovation.

**Figure 13:**
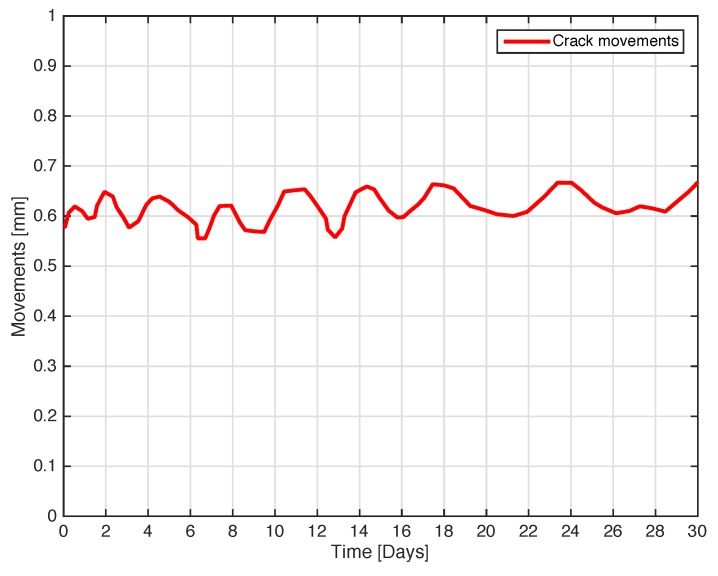
Crack movements vs. time. The tag placed in position A in the west side of the building.

**Figure 14:**
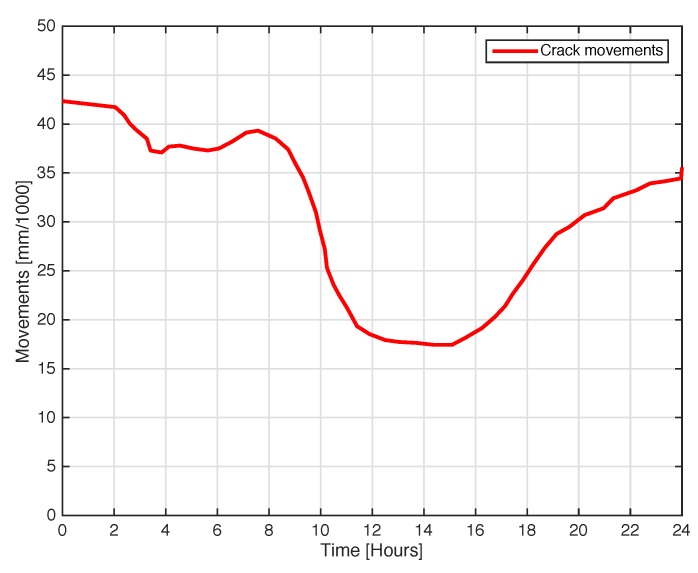
Crack movements vs. time. One day measurement showing the small movement due to temperature changes. Tag placed in position A.

**Figure 15:**
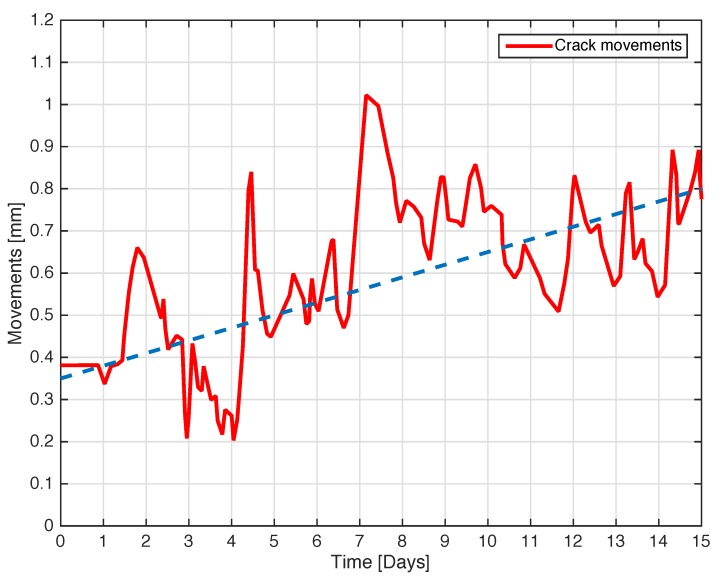
Crack movements vs. time. The tag placed on position B in the north side of the building, showing a dangerous situation with possible collapse.

**Figure 16:**
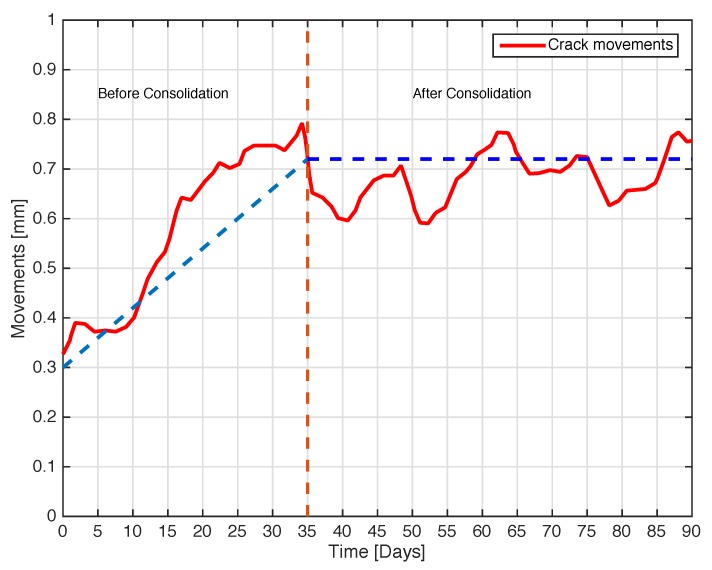
Crack movements vs. time. The tag placed on position B in the north side of the building, before and after the consolidation.

The next experiment concerns a measurement campaign performed in a real scenario. In particular, a 110-year-old building under renovation has been considered. The structure presents three suspicious cracks, two of them are located on the north side of the building and one on the west side. In the experimental scenario, three different tags have been considered and placed in the positions A, B, and C indicated in Figure 8. In particular the tag placed in position A was equipped with the VCO while the tags placed in positions B and C are provided with a microcontroller, and they operate with the EM4102 protocol and the Manchester modulation. The reader and the tags have been aligned in line of sight. The alignment has been done with a laser lever and the distance measured with a laser telemeter. However, it is worth noticing that a perfect alignment is not mandatory thanks to the circular polarization. Load-bearing walls of the building are composed by a mixture of different river rocks, the heterogeneous mix of material makes the crack particularly insidious, and a monitoring campaign is mandatory to prevent sudden collapses. A photo of the considered scenario indicating the position of crack is shown in Figure 7. Three MST tags have been placed to monitoring the cracks movements. The photos of the MST tag prototypes placed on location A and B are reported in Figure 9 and Figure 8 respectively. As it can be noticed the MST probes have been position across the cracks and the two patch antennas, which also act as anchor points have been fixed across the crack flaps. It is worth noticing that MST tags are insensitive to the environment and it was widely demonstrated that MST tags can operate in no-farfield and no-freespace scenario [24,25,29]. Thanks to the circular polarization of the antenna reader the MST tags are quite insensitive to the crack orientation. This is demonstrated in the following experiment where an MST tag communication capabilities has been assessed. In particular Figure 10 reports the considered experimental setup. Figure 11 reports the measured signal power versus different tag orientations. As it can be noticed from the data of Figure 11 the signal is quite stable. The considered operative range was 5 m. The measurement campaign has been completed in about four months. In the following, the obtained results are summarized. The first data set, concerns one month measurement campaign, where a MST probe has been placed to monitoring the crack located in the west side of the building at position called Tag A, with reference to Figure 12. The reader has been placed at a distance D=5 m, the data measured by the MST probe has been continuously collected and stored by the reader every half second to monitoring the effects of sudden collapses due to vibrations. The data are reported in Figure 13, as it can be noticed the crack movements were very limited, a maximum displacement of about $d_{s}=0.06$ mm has been detected during one month of measurements demonstrating the stability of the crack and a reduced risk of collapse of the upper west side of the building. The high amount of data permits a detailed analysis of the material behaviour, in particular Figure 14 reports a zoomed version of the data collected during a summer day, the data reported in Figure 14 show a maximum displacement of about $d_{s}=0.0020$ mm probably due to temperature excursion which produce a dilatation of the material. Data collected by the MST tag placed in the north side of the building and aimed at monitoring the crack are reported in Figure 8. Considering a direct visual inspection the crack reported in Figure 8 seems to be less critical with respect to the damage reported in Figure 9 because the distance between crack flaps is limited. The data reported in Figure 15 clearly demonstrate the dangerous characteristics of crack placed in the north side of the building in position B, reference Figure 12. In particular after only fifteen days, the crack flaps reach a displacement of more than half millimeter with a suspicious increasing trend. The company in charge for the building renovation, considering the obtained data provided to strengthen the damaged wall with a reinforced concrete structure in order to prevent the north side upper section collapse of the building. Figure 16 shows the crack displacements before and after the consolidation operation. As it can be noticed the data reported in Figure 16 clearly demonstrated the efficacy of the wall consolidation.

**Figure 17:**
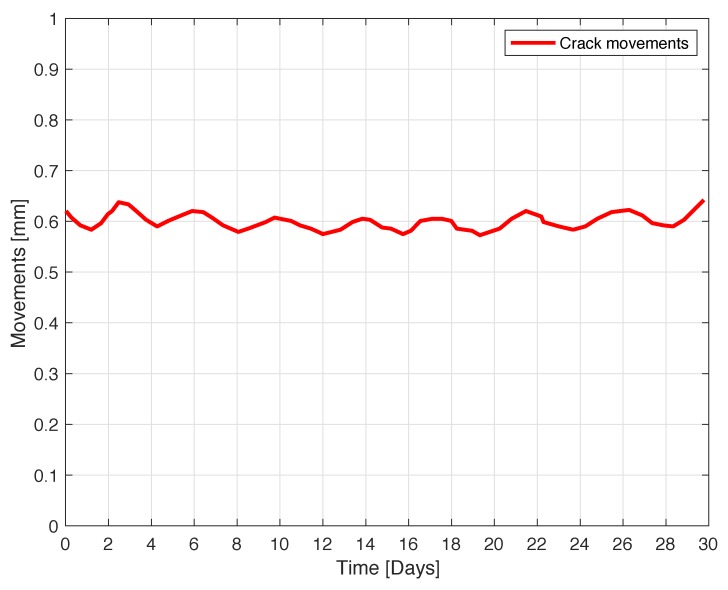
Crack movements vs. time. The tag placed on position C in the north side of the building, showing a safe situation.

The data collected by the MST probe placed in position C, reference Figure 12 and reported in Figure 17 revealed a non dangerous crack, in particular the crack flaps moved for a maximum of about $d_{s}=0.04$ mm after one month of monitoring campaign with a stable trend.

## 4. Conclusions

A system for the real time monitoring of cracks in civil engineering structures has been presented, fabricated and experimentally assessed. The system is based on the MST technique in order to obtain an effective operative range of meters. The MST sensor tags are composed by two rectangular patch antennas, a rectifying antenna sub-circuit, a piezoelectric sensor able to detect millimetric movements of cracks, and a transmitting antenna able to deliver the information toward a wireless channel at 10 GHz. The system has been assessed considering a 110 years old building under renovation. The obtained results were quite promising and they demonstrated the effectiveness of the proposed MST-RFID based system.
